# vTrust: An IoT-Enabled Trust-Based Secure Wireless Energy Sharing Mechanism for Vehicular Ad Hoc Networks

**DOI:** 10.3390/s21217363

**Published:** 2021-11-05

**Authors:** Kamran Ahmad Awan, Ikram Ud Din, Ahmad Almogren, Byung-Seo Kim, Ayman Altameem

**Affiliations:** 1 Department of Information Technology, The University of Haripur, Haripur 22620, Pakistan; kamranawan.2955@gmail.com (K.A.A.); ikramuddin205@yahoo.com (I.U.D.); 2 Chair of Cyber Security, Department of Computer Science, College of Computer and Information Sciences, King Saud University, Riyadh 11633, Saudi Arabia; 3 Department of Software and Communication Engineering, Hongik University, Sejong 30016, Korea; 4 Department of Natural and Engineering Sciences, College of Applied Studies and Community Services, King Saud University, Riyadh 11543, Saudi Arabia; aaltameem@ksu.edu.sa

**Keywords:** vehicular ad-hoc network, security, threat traceability, trust management, energy resources, trustworthiness

## Abstract

Vehicular Ad hoc Network (VANET) is a modern concept that enables network nodes to communicate and disseminate information. VANET is a heterogeneous network, due to which the VANET environment exposes to have various security and privacy challenges. In the future, the automobile industry will progress towards assembling electric vehicles containing energy storage batteries employing these resources to travel as an alternative to gasoline/petroleum. These vehicles may have the capability to share their energy resources upon the request of vehicles having limited energy resources. In this article, we have proposed a trust management-based secure energy sharing mechanism, named vTrust, which computes the trust degree of nodes to authenticate nodes. The proposed mechanism is a multi-leveled centralized approach utilizing both the infrastructure and vehicles to sustain a secure environment. The proposed vTrust can aggregate and propagate the degree of trust to enhance scalability. The node that requests to obtain the energy resources may have to maintain a specified level of trust threshold for earning resources. We have also evaluated the performance of the proposed mechanism against several existing approaches and determine that the proposed mechanism can efficiently manage a secure environment during resource sharing by maintaining average malicious nodes detection of 91.3% and average successful energy sharing rate of 89.5%, which is significantly higher in comparison to the existing approaches.

## 1. Introduction

Vehicular Ad hoc Network (VANET) [1] is introduced to overcome the restraints of road and driving difficulties occurred due to blind spots [2] wherein traffic information system (TIS) [3], two-tier TIS [4], and emergency systems [5] help significantly to overcome these challenges. VANET is a modification of mobile ad hoc network (MANET) [6] that provides vehicle-to-vehicle (V2V) [7] and vehicle-to-infrastructure (V2I) communications [8]. The VANET environment is also equipped with intelligent transportation system (ITS) [9] that provides protocols to communicate with network nodes [10]. To reduce the effect of vehicle smoke on climate, the automobile industry progressing towards electric vehicles. Various top companies have already introduced these vehicles where the most popular companies manufacturing electric vehicle are Tesla, General Motors, and Nissan. It means that in the near future, electric vehicles will become the major mode of traveling [11]. After the successful implementation of VANET, the network participating nodes will be equipped with electrical batteries to drive their engines. In addition, charging points may be provided for these batteries at nearby positions [12]. Further, energy resources in vehicles can only contain limited energy and are required to be recharged time-by-time [13]. During long traveling, charging a battery and finding a charging spot can be unmanageable in unfamiliar and remote territories. As an alternative, the automobile companies will have to introduce such a wireless energy resource sharing mechanism wherein static/mobile vehicles should share/receive resources with each other to address the challenge of limited energy storage resources [14,15].

**Figure 1:**
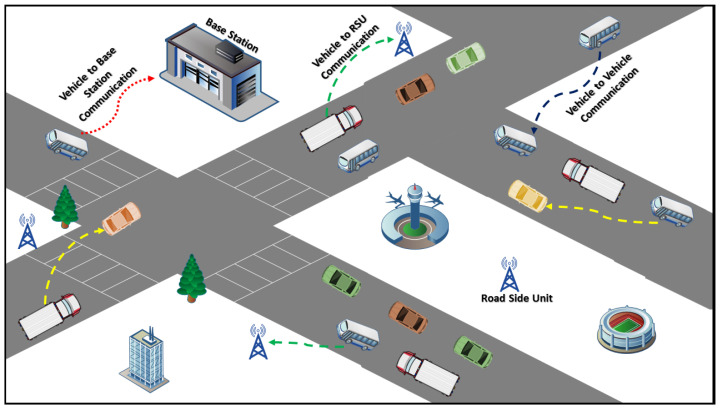
The VANET architecture.

The communication in VANET is independent [16] where one vehicle can transmit and acquire information from other vehicles and infrastructures [17], as shown in Figure 1. Independence in communications mounts several challenges and vulnerabilities [18], and provides an easy pathway to malicious and compromised nodes for administering potential attacks, such as denial-of-service (DoS) and sybil attacks [19,20,21]. Initially, the process of energy sharing begins with the requested messages, which can further enhance the vulnerabilities of the VANET environment. To address the challenges of energy resources, we have proposed a trust-based mechanism, which utilizes the components of trust for evaluating the trustworthiness and authenticity of vehicles so that a secure environment is maintained. The proposed mechanism is also capable of identifying nodes generating requests while having enough resources by restricting them to generate and broadcast requesting messages to neighboring nodes. The proposed mechanism evaluates the trust of nodes based on three parameters, i.e., ability, benevolence, and integrity. To evaluate the ability of a trustee or trustor, vTrust utilizes the stability trust parameter. The proposed mechanism is a multi-leveled centralized trust management approach wherein roadside units (RSUs) [22] and base units [23] have been utilized to propagate and aggregate the trust degree. The RSUs also have the capability to communicate with neighboring RSUs for the purpose of sharing trust computations of particular nodes when required. To date, no mechanism is proposed to secure the process of energy sharing. The novelty of the proposed mechanism can be summarized as follows:

- A three-tier trust management-based computational approach is proposed to maintain security during energy sharing.
- It has the ability to evaluate direct and indirect trust whereas no extra infrastructure is required.
- It has the potential to propagate and aggregate trust for combining the current and previous trust to improve scalability and eliminate vulnerabilities of on-off attacks.

The structure of the rest of the article is as follows: Section 2 discusses the existing security challenges and approaches associated with energy sharing. Section 3 elaborates the proposed approach along with its architecture in Section 3.1. Section 3.2 specifically represents trust parameters along with direct and indirect evaluation processes of the vTrust. Section 4 elaborates the simulation outcomes and discusses the comparative analysis of the proposed mechanism with the existing ones. Finally, Section 5 concludes the article.

## 2. Literature Review

It is significant to address the challenges associated with a secure energy sharing process because malicious and compromised nodes can generate a request to get energy from neighboring nodes. However, no notable work has been proposed till date to address the security challenges of energy sharing in electric vehicles. This section elaborates VANET approaches proposed for maintaining security, which also examine the limitations to achieve an adequate secure environment (see Table 1).

The concept of wireless energy sharing is discussed in [29], where the authors stated that the energy crowd-sharing framework can be successfully implemented by addressing the associated challenges. The study divides the challenges into three dimensions, i.e., crowd-sharing, enabling technologies, and deployment. The enabling technologies include the challenges associated with energy-storing batteries such as limited capacity [30], insufficient computing ability, energy deprivation, and battery health. The energy crowd-sharing includes challenges of security [31], privacy [32], trust [33], and reliability of energy [34]. In [35], several security and privacy issues have been identified related to VANET where various attacks, e.g., DoS [36], bandwidth consumption [37], and jamming attacks [38], are significant challenges. Furthermore, the proposed study also furnishes five key properties, i.e., decentralized trust management [39], scalability [40], privacy, robustness, and information sparsity.

In [24], a trust-based security scheme for message exchange (TSME) is proposed to address the security and privacy challenges linked with message interchange between nodes. Blockchain [41] is one of the most prominent solutions to maintain security by forming a chain of blocks along with a hash encryption [42] to maintain the integrity of data. The study in [25] utilizes a similar idea and merges it with a trust mechanism to prevent malicious and compromised nodes from joining the environment and communicating with neighboring nodes.

In 2019, a trust management mechanism was proposed, which focuses on managing trust based on communities [43]. The proposed study illustrates that IoT consists of numerous heterogeneous devices, e.g., home appliances [44], smart gadgets, etc. [45]. The self, green, social, and QoS trust (SGSQoT)  [26] utilizes the concept of community architecture to manage trust among nodes. The novelty of the proposed scheme is such that it uses self, social, and green properties of trust to maintain resilience towards attacks.

Another mechanism, named trust management system based on communities of interest for the social IoT (TMCOI-SIoT) [27], is proposed that focuses on maintaining trust in the social IoT. The novelty of the proposed mechanism is the utilization of the Kalman filter technique [46] to predict the behavior of nodes and to prevent the on-off attacks. The proposed architecture is based on a community of trust and divides the IoT nodes based on their common interest. The study focuses on preventing on-off attacks using Kalman filtering technique [47], however, its performance needs to be validated through extensive evaluation against several other potential attacks, such as false positive and false negative, good and bad-mouthing attacks.

The trust management mechanism for service-oriented architecture (SOA-TM) is proposed in [28], which focuses on maintaining trust among nodes. In the proposed approach, the nodes are connected with a dedicated network. At the user level, the owner identifies the centralized devices to store the trustworthiness and feedback. At the central level, devices maintain and manage the collective score of trustworthiness. The significant aspect of SOA-TM is to obtain chromosomes of two or more parents and cross-over using a genetic algorithm. However, the system’s performance requires validation through extensive simulations, which must include potential attacks. Several other energy-based systems have been proposed in the literature, which can be found in [48,49,50,51,52,53].

## 3. Proposed vTrust Mechanism

The VANET environment is heterogeneous where nodes can join and leave the network at any time. The concept of energy sharing raises several security challenges related to identification of malicious and compromised nodes. Therefore, it is significant to address those challenges to provide secure sharing of resources. The proposed vTrust mechanism addresses the security challenges and provides a trust-based approach to identify such a node that attempts to execute attacks for gaining advantages.

### 3.1. Architecture of vTrust

The vTrust mechanism utilizes the components of trust to evaluate the level of trustworthiness and authenticity of particular nodes for sharing energy resources concerning trust level. To receive energy resources, the nodes require to generate a request to the neighboring nodes, whereas the request generated by the node also contains information of the current level of energy resources as an authenticity. The driver of requesting nodes can only broadcast seeking requests, and he/she cannot have access to alter the generated message. When neighboring nodes receive the request and accept to share energy resources, then that requesting vehicle automatically generates an informative message to other nodes. Hence, other nodes do not accept the request to share resources. To reduce the overhead ratio, the proposed approach restricts the seeking request by delivering the message only to those nodes which declare themselves as volunteer to share their resources. The RSUs also maintain a table of nodes with rich energy resources. In case of seeking request, the RSU provides information to reduce the number of broadcast messages generated by resource seeking nodes. The trust parameters utilized by vTrust include ability, benevolence, and integrity. To increase the security and effectiveness of the proposed approach, the vTrust utilizes both direct and indirect trust evaluations where RSUs provide observations in both scenarios.

The only assumption made in the proposed mechanism is that all VANET nodes are IoT-enabled and can have the capability to store information and process data. The proposed mechanism is an event-based process, which means that vehicles only evaluate the trust degree when they receive the energy sharing request or when a particular node accepts to share resources. To propagate and aggregate the trust degree, nodes are required to request a nearby RSU. If the RSU contains the previous trust degree, then it will directly transmit the value. Otherwise, it generates the request to the neighboring RSU for obtaining the previous trust degree about a particular node. To reduce the communication burden, the vTrust approach does not allow nodes to generate energy receiving requests to the nodes having enough energy resources. The threshold is elaborated in Section 3.3. Furthermore, to increase the success rate of energy sharing, the proposed approach also prioritizes the request generated by needy nodes, for example, in case the vehicle is parked, then the generated request will only be received by the nearby parked nodes; whereas when nodes are moving on highways, then the generated request and sharing of energy resources will only be received by mobile nodes.

**Figure 2:**
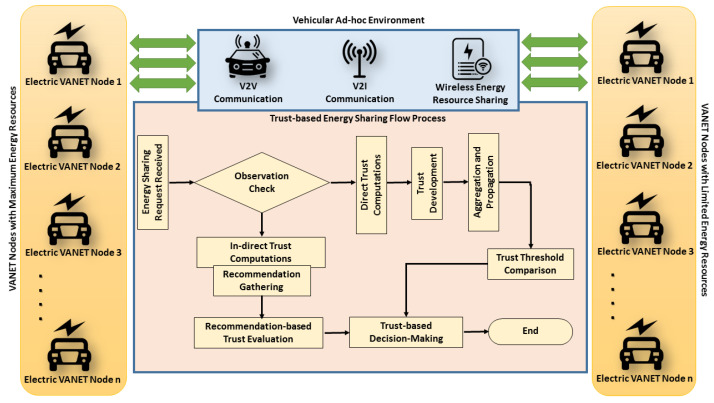
The architecture of the proposed vTrust mechanism.

**Figure 3:**
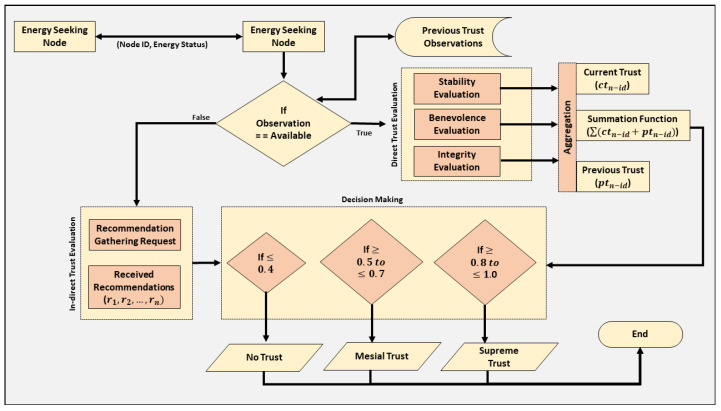
The workflow diagram of vTrust mechanism.

The architecture of the proposed trust management mechanism consists of several modules, i.e., nodes with maximum and minimal energy resources, and wireless energy sharing module along with integrated trust-based evaluation process, as illustrated by Figure 2. The workflow of evaluation is represented in Figure 3. In the proposed architecture, the VANET nodes will declare themselves as volunteer based on the excess energy resources. The energy is shared upon the request of a node that requires resources. Thus, resource seekers have to pay incentives electronically against the amount of energy they receive from the volunteer nodes. The architecture also contains both the facility, i.e., V2V and V2I communications, so that nodes can use the V2V facility to communicate with neighboring one and V2I capabilities to communicate with infrastructures in case they require to gather recommendation against any communicating node. The major assumption in the proposed architecture is that all the nodes are IoT-enabled and can store, process, communicate, and keep track of energy resources against their destinations and upcoming charging points. The architecture also includes the evaluation of trust based on direct and indirect evaluations, as discussed in Section 3.2. The process of energy sharing starts from neighboring nodes when a node requires energy in case of emergency. The energy-seeking node will broadcast its request towards all adjacent nodes and will wait for other nodes to respond. When a node with sufficient energy resources responds, the transmitted request will be terminated automatically by the on board units. Based on the predefined incentives, the seeker node will pay the incentive to the node that provides the energy resources.

### 3.2. Trust Parameters and Evaluation

The trust parameters utilized in the proposed approach have been selected considering three important factors: computational reduction, efficient resource utilization, and energy efficiency. The trust parameters utilized in vTrust include ability, benevolence, and integrity. The workflow is presented in Figure 3 and pseudocode is illustrated by Algorithm 1. The ability and benevolence parameters belong to the reputation component of trust whereas integrity belongs to the knowledge component. The parameters of reputation components help the network participating nodes to evaluate the stability and benevolence of a requesting node. Similarly, the parameters utilized related to knowledge will let the nodes evaluate the degree of honesty of a particular node. Both the evaluations help energy providing node to decide whether it is safe to share the resources or not.
**Algorithm** **1** Degree of Trust Computation Flow Process1:**procedure**Observation Gathering($D_{ob}$)2:    Perceive Previous Available Observation3:    **if** (Observation are available) **then**4:        Compute Direct Trust;5:    **else**6:        Compute Indirect Trust;7:**procedure**Direct Trust Evaluation($D_{trust}^{n-id}$)8:    Ability Evaluation as Equation (1)9:    Benevolence Evaluation as Equation (2)10:    Summation to Reputation Trust Degree as Equation (3)11:    Integrity Evaluation as Equation (4)12:    Absolute Trust Evaluation as Equation (5)13:**procedure**Indirect Trust Evaluation($ID_{trust}^{n-id}$)14:    Recommendation Gathering as Equation (6)15:    Applying Summation to received recommendation as Equation (7)16:**procedure**Direct Trust Aggregation($T_{abs}^{dev}$)17:    Aggregation Computation as Equation (8)18:**procedure**Decision Making(ϕ(t))19:    Condition for Decision Making as Equation (9)20:    Valid Node if Trust ≥ 0.5.21:    Exit

The process of trust evaluation is divided into two types: direct trust evaluation and indirect trust evaluation. In direct trust evaluation, nodes utilize the pre-discussed parameters to evaluate the degree of trust. While in the indirect trust evaluation, the nodes generate requests to gather recommendations regarding a particular node from neighbors or in some situations may be from RSUs. As VANET is heterogeneous due to its mobility nature, it is critical to consider and provide adequate capability to the network nodes for accurate indirect evaluation. To begin with direct trust evaluation, the node searches for observations in the stored data. If observations are available, then these are utilized by the node to evaluate direct trust, else node will rely on indirect trust evaluation. The symbols used in the trust evaluation process are illustrated in Table 2. When the observations are available, the nodes evaluate the ability of other node, i.e., stability to provide services, and the computation is performed by the node, as illustrated by Equation (1).

(1) $$T_{ab}^{rep}=\lim_{1.0}\left[\sum (ab_{p\to r}^{rep_{1}}+ab_{p\to r}^{rep_{2}}+\ldots +ab_{p\to r}^{rep_{n}})\right]$$

In Equation (1), *T* represents trust computation, *ab* shows the trust degree of a particular node in terms of ability, *rep* represents the reputation components of trust, *p* and *r* exhibit the trustor (resource rich node), and trustee (resource seeking nodes), respectively. Whereas $rep_{1}\ldots rep_{n}$ represents the number of available observations. After the evaluation of ability, the nodes then evaluate the benevolence parameter by gathering available observations, as represented by Equation (2).

(2) $$T_{be}^{rep}=\lim_{1.0}\left[\sum (be_{p\to r}^{rep_{1}}+be_{p\to r}^{rep_{2}}+\ldots +be_{p\to r}^{rep_{n}})\right]$$

In Equation (2), *be* represents the benevolence trust degree, which is evaluated based on the available observations; *p* shows the nodes with rich resource; and *r* exhibits the nodes generating energy seeking requests. After calculating the reputation parameter, vTrust applies the summation function to evaluate the absolute degree of trust, as shown in Equation (3).

(3) $$T_{p\to r}^{rep}\left[ab⊎be\right]=\sum_{0.0}^{1.0}\left[T_{ab}^{rep}+T_{be}^{rep}\right]$$

The computation of absolute reputation value within the limit leads the evaluation to knowledge computation that has been evaluated based on the integrity parameter, which shows the persistence of a particular node. The evaluation of integrity is shown in Equation (4).

(4) $$T_{ie}^{kno}=\lim_{1.0}\left[\sum (ie_{p\to r}^{kno1}+ie_{p\to r}^{kno2}+\ldots +ie_{p\to r}^{knon})\right]$$

In Equation (4), *ie* represents the integrity evaluation of nodes, *p* is the trustor node, and *r* shows the trustee node. The direct trust evaluation is only possible when nodes contain the previous observations. In case the previous observation is not available, then nodes have to rely on the observations received after requesting the nearby RSUs. The observation received by the RSUs is also considered as direct observation. In case RSUs do not have observations, then the nodes request the neighbors, which is considered as indirect trust. After the evaluation of knowledge component, the vTrust evaluates the absolute trust degree of a particular node by applying summation to the pre-evaluated reputation and knowledge components (see Equation (5)).

(5) $$trust_{p\to r}^{ab}=\sum_{0.0}^{1.0}\left[T_{p\to r}^{rep}\left(ab⊎be\right)+T_{ie}^{kno}\left(⊎ie\right)\right]$$

Equation (5) provides the degree of trust of a particular node based on which the resource provider decides whether they want to share their resources or not. The decision-making process is elaborated in the coming section. In Equation (5), *ab, p, r* represent absolute, trustor, and trustee, respectively. Further, *ab* represents reputation evaluation of trust degree and *ab*, and *be* are the ability and benevolence. The *kno* represents knowledge evaluation of trust degree and *ie* is the trust degree of integrity. The nodes utilize the pre-defined parameters only when the observations are available, but if the required observations are unavailable, then a node will request the nearby RSUs to gather the observation. In case the observations are not available with the nearby RSUs, then these RSUs can request the prior located RSUs. When nodes gather observations from RSUs and utilize them to evaluate the degree of trust, this process is known as indirect trust-evaluation or recommendation-based evaluation. When nodes receive the observations, then the trust is evaluated, as illustrated in Equations (6) and (7).

(6) $$t_{p\to r}^{rec}=\sum \left(s_{rsu_{1}}^{ob_{1}}+s_{rsu_{1}}^{ob_{2}}+\ldots +s_{rsu_{1}}^{ob_{n}}\right)$$

(7) $$T_{p\to r}^{abs}=\sum_{0.0}^{1.0}\left[t_{p\to r}^{rec}\right]$$

In Equation (6), $t_{p\to r}^{rec}$ represents the trust evaluation of node *p (trustor)* towards *r (trustee)* and *rec* represents the recommendation-based evaluation. Further, *s* represents source RSUs and $ob_{1}$ is the number of observations received. Equation (7) shows the computation of absolute trust within the threshold value, whereas *p* represents trustor, *r* shows trustee, *abs* is the absolute trust, and *rec* exhibits the recommendations received by the trustor.

### 3.3. Aggregation, Threshold and Decision Making

The vTrust approach can also store and utilize the trust degree whenever required in the future. For aggregation, the vTrust attains the previous trust degree and aggregates it with the current evaluation during direct and indirect trust evaluation. The process of trust aggregation is shown in Equation (8).

(8) $$T_{p\to r}^{agt}=trust_{p\to r}^{ab}+t_{s_{id}}\left[pt_{s_{id}}^{ob_{1}}+pt_{s_{id}}^{ob_{2}}+\ldots +pt_{s_{id}}^{ob_{n}}\right]$$

Equation (8) represents the evaluation of the direct trust aggregation process in which the number of previous trust evaluation is aggregated with the current trust to evaluate the aggregated absolute trust for achieving the scalability and reducing the chances of successful execution of whitewashing attacks. In Equation (8), *agt* is aggregated trust, *p* shows resource provider, *r* represents resource seeking node, and *pt* is the past trust degree of a particular node represented by $s_{id}$, while $ob_{1}\ldots ob_{n}$ shows past observations. The threshold comparison of trust is a significant process that provides the capability to compare the absolute trust degree with the predefined threshold to decide either a node is trustworthy or not.

In vTrust, the range of trust degree is 0.0–1.0, which means all the evaluated trust is ranked between these values, whereas this range is further categorized into several characters to identify the level of trustworthiness. The range of trust between 0.0 and 1.0 is selected to reduce the computational burden and storage memory by which nodes are able to perform better with less burden. The range of trust is predefined that helps nodes to decide whether the communicating node is trustworthy or not. The conditions of decision making based on predefined threshold values are illustrated by Equation (9). The trust range is divided into four parts for nodes classification based on their computed degree of trust. Moreover, the final value of computed trust will be round off value that follows the rule of rounding-up and rounding down. In rounding-up, the value is rounded up to the next numeric digit if it is >5 and rounded down if it is ≤5. The proposed approach assigns 0.5 as the default degree of trust, and it is assigned by the RSUs whenever nodes join the network. Further, 0.0–0.4 is classified as no-trust, which means that if nodes own the trust degree between these values, then resource providers will not be able to communicate and share their resources with the requesting nodes. Moreover, 0.5–0.7 is a middle trust degree. If nodes contain 0.5–0.7, then the resource owners decide to transfer resources of their own choice. Similarly, nodes with higher degree of trust, i.e., 0.8–1.0, will get the maximum amount of resources they need considering the resources a particular owner wants to share. Thus, those nodes who share these resources will get incentives in the shape of free-charge from the energy or financial gain such as free service to their car, etc.

(9) $$\phi \left(t\right)=\left\{\begin{array}{c} Supreme-Trust\&\;\mathrm{if}\;t\geq 0.8\\ Mesial-Trust\&\;\mathrm{if}\;t\geq 0.6\\ Default-Trust\&\;\mathrm{if}\;t\overset{\underset{\mathrm{def}}{}}{=}0.5\\ No-Trust\&\;\mathrm{if}\;t\leq 0.5 \end{array}\right.$$

## 4. Simulations and Experimental Results

This section discusses the simulation outcome of vTrust and other existing approaches, i.e., TMCOI-SIoT [27], SOA-TM [28], and SQS-QoT [26], to validate the performance. The simulation setup is illustrated in Table 3, which is utilized under different scenarios and distinct potential attacks. The simulator used to simulate the proposed approach is objective modular network (OMNeT++) along with simulation of urban mobility (SUMO) to implement and evaluate the approaches in real-world scenarios. The urban area simulations are performed in Islamabad Capital Territory (33.6938118, 73.0651511) of Pakistan. The dimensions of the selected map used for the simulation is x = 33.6994, 33.6716 and y = 72.9749, 73.0515, while the rural area simulation is performed in Haripur district (33.9944889, 72.9331737) of Pakistan whereby the selected map’s dimensions are x = 34.0019, 33.9936 and y = 72.9269, 72.9384. The major focus of simulations is to evaluate the ability of approaches to maintain security before sharing energy resources. In addition, the identification and elimination of malicious or compromised nodes is the primary objective. The range of degree of trust is 0.0 to 1.0, whereas the rest of the classification is explained in Section 3.3.

### 4.1. Malicious Node Detection

It is significant to detect the malicious and compromised nodes with maximized accuracy, as if nodes failed to identify such nodes, then it increases the risk of false sharing that can cause energy wastage. To maintain and securely transfer the energy resources, it is significant to identify and eliminate the node that generates false request intentionally or gets compromised to become resource seekers. Here, in this section, three different scenarios have been implemented to evaluate the comparative performance of vTrust with other approaches.

**Figure 4:**
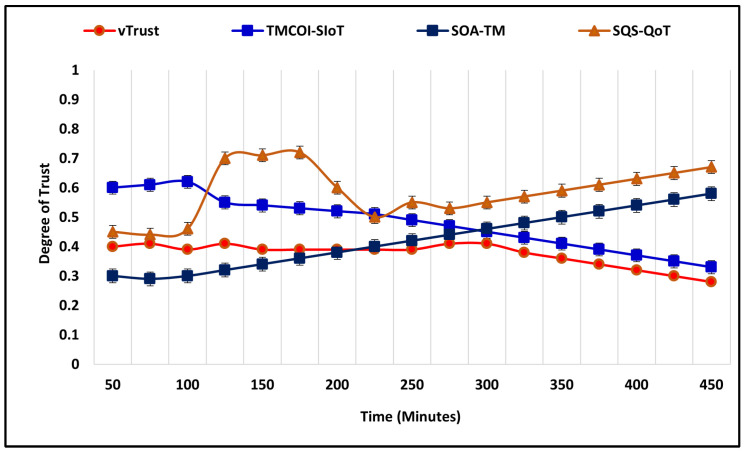
Malicious nodes detection with 50 nodes.

In the first scenario, the number of nodes is 50, simulation time is 450 m, range of trust is 0.0–1.0, and the percentage of the malicious node is 40%. In the beginning, vTrust allocates the default degree of trust, i.e., 0.5, whereas with the time as it collects the observations, the approach starts assigning no-trust which is below 0.5. In comparison, the performance of TMCOI-SIoT increases significantly but takes more time and starts the allocation of no-trust after 220 m. The notable aspects of fluctuation in the increase and decrease of simulation outcomes is due to the fact that when VANET nodes are able to identify malicious nodes, then the outcome of average trust degree will be low, which depends on the malicious-to-valid nodes percentage ratio. If the percentage ratio of malicious nodes is higher and the existing approaches are able to identify these nodes successfully, then the average trust degree must be low, i.e., <0.5. Moreover, if the approaches are not able to accurately identify malicious nodes, then they will assign them higher degree and label them as valid nodes. On the other hand, the average trust degree will become higher with the expense of increased security vulnerabilities. The comparative performance analysis is illustrated in Figure 4.

**Figure 5:**
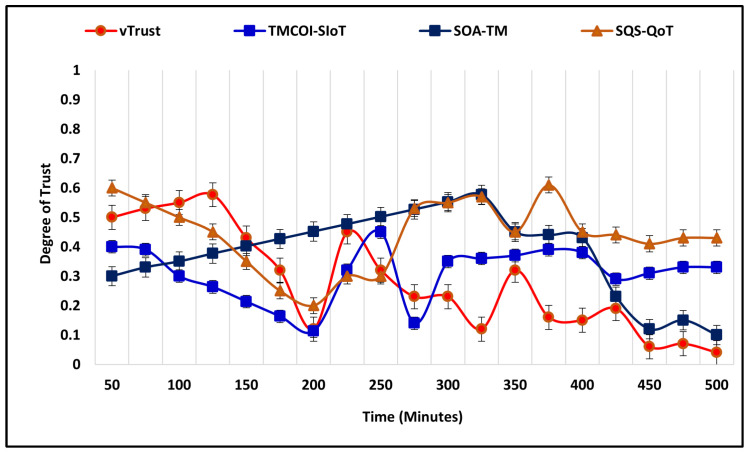
Malicious nodes detection with 100 nodes.

In the second scenario of malicious node detection, the number of nodes is 100, the simulation time is 500 m, range of trust is same as previous, whereas the percentage of malicious nodes is increased to 60%. The simulation outcome is illustrated by Figure 5 that shows the effective performance of vTrust beginning with a decline to the average degree of trust from 0.5 to 0.2. It shows the enhanced performance in terms of identification of malicious nodes by assigning a low degree of trust. In comparison, SoA-TM also performs significantly commendable with the expense of time, as Figure 5 shows a decline in the trust degree after 125 m and continuous increase in the degree of trust from start to 325 m.

**Figure 6:**
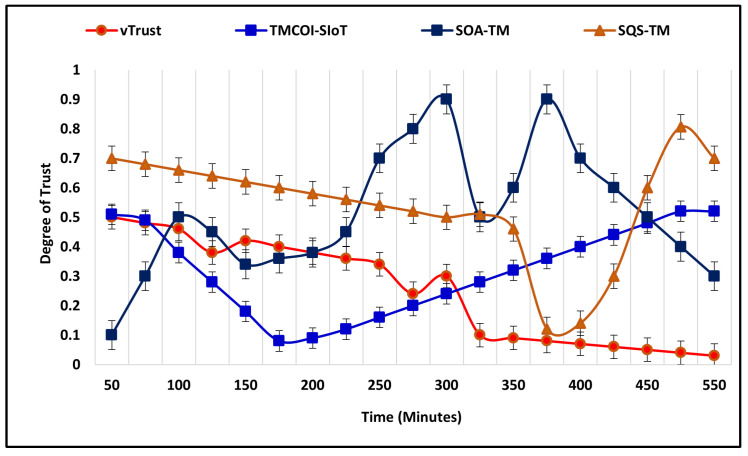
Malicious nodes detection with 150 nodes.

To further extend the performance validation of vTrust, we have designed the third scenario by increasing the number of nodes to 150, simulation time 550 m, malicious node percentage to 80%, and trust ranging from 0.0 to 1.0. The increase in malicious nodes has effected the identification of these nodes. However, vTrust has effectively maintained and provided stable performance, as illustrated by Figure 6. In comparison, existing approaches face difficulties to identify malicious nodes due to higher number of malicious nodes and assigning higher trust degrees.

### 4.2. Rate of Successful Energy Sharing

This section illustrates the energy sharing between resource seeker and provider after the evaluation and decision making as sharing of resources completely depends on the trust degree. The simulation of successful energy sharing is performed by implementing the urban and rural areas with different average node speeds. In urban areas, nodes’ average speed lies between 60 and 90 km/h, whereas in rural areas, this speed is between 40 and 60 km/h. The energy shared between nodes is measured in Joule (J), whereas the simulation time is set as 550 m. The total number of nodes is 150, whereas these are further divided based on their roles. Furthermore, malicious node distribution is different in urban and rural areas, i.e., initially the malicious percentage in urban areas is 60%, which reaches 75% after 250 m. Whereas in rural areas at the beginning, the node percentage is 40%, which is increased by 20% after 300 m. Further, it is also significant to evaluate the performance on potential attacks. Thus, the malicious percentage of nodes is further divided into different groups based on attacks, such as sybil attack, on-off attack, white-washing attack, and false-seeking request. Higher energy sharing depends on QoS provided by the resource provider towards the seeker and also the identification of malicious nodes plays an essential role to save the sharing of false energy.

**Figure 7:**
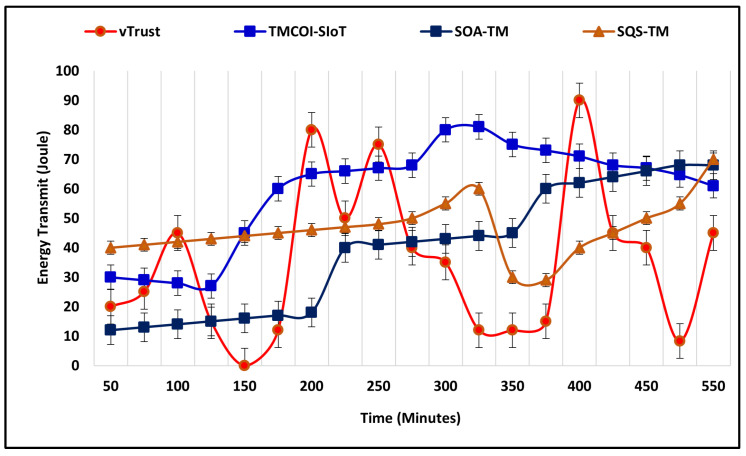
Average energy shared in rural environment.

**Figure 8:**
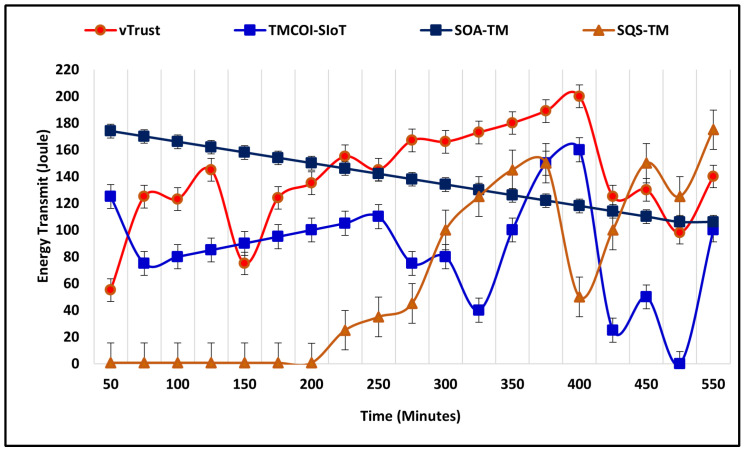
Average energy shared in urban environment.

Figure 7 represents the comparative performance of vTrust with the existing approaches. The vTrust in rural environments performs significantly by sharing higher amount of energy at different time intervals, such as 180 m, 250 m, and most importantly at 410 m. The TMCOI-SIoT also maintains a notable performance by sharing the consistent energy resource between 160 m and achieves higher amount of sharing at 315 m. Moreover, the same scenario of rural area has been implemented to evaluate the sharing of energy resources, but the average speed of nodes is now reduced to 40–60 km/h, which may reduce the mobility challenges faced by nodes in rural areas due to higher average speed. Figure 8 represents the sharing of energy between nodes and represents that the reduction in speed increases the success rate and energy shared towards seekers in urban areas.

### 4.3. Energy Consumption of Trust Computations

**Figure 9:**
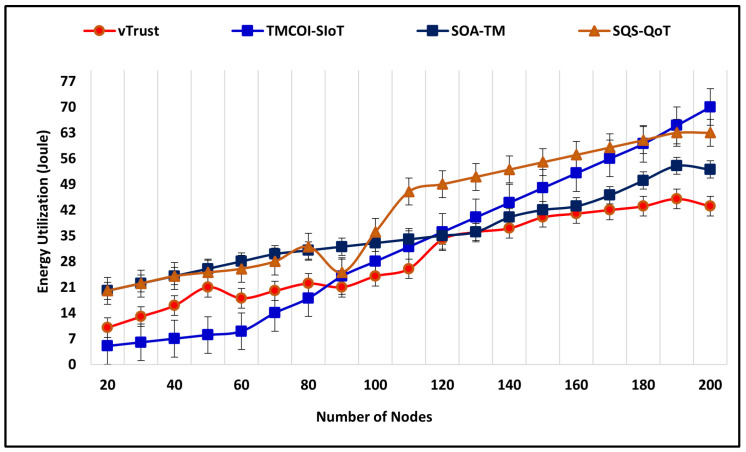
Average energy consumed to compute trust.

In the future, it becomes significant to maintain a green environment by utilizing energy resources efficiently. This section illustrates the average energy consumption of vTrust as compared to the existing approaches. The energy utilized by these approaches is measured in Joule (J) and the simulation time is 550 m. At the beginning of simulations, the number of nodes is 20, which increases by 20 nodes every 20 m. Figure 9 represents the rate of energy consumption of vTrust in comparison to existing approaches and the outcomes of simulation confirm that vTrust is energy efficient even when the number of nodes increases from 120 to 200. In addition, SOA-TM also performs effectively to reduce the energy consumption. The simulation results show the prompt elevation of TMCOI-SIoT and utilizes the maximum amount of energy.

## 5. Conclusions

The sharing of energy among nodes is a novel and effective concept to depreciate the concerns faced by electric vehicles on highways or in remote areas where recharge stations are not available. Due to large geographical areas, it is impractical to provide a recharge station at a favorable distance for each vehicle’s convenience. Besides, if manufacturing companies provide the recharge station, yet sharing of energy during mobility impersonates a vital role as it saves time of the owners. However, it may increase vulnerabilities as nodes with rich resources can also generate a false request. Considering such circumstances, it becomes imperative to maintain the security between resource seekers and providers. In this article, a trust-based energy sharing approach is proposed for the identification of malicious and compromised nodes to maintain a trustworthy environment, where nodes can communicate and share resources among seekers who own higher degree of trust. The proposed approach is a lightweight approach that performs computations using trust parameters to evaluate trust degree and aggregates the computed value with the previous trust degree to decide whether nodes are trustworthy or not. The simulation outcome also exhibits the effective performance of the proposed approach in comparison with the existing ones with the maximum malicious nodes identification rate, maximum energy sharing, and reduced energy computation to perform trust computations. The proposed work can be extended by utilizing the experience propagation using a base station with the prediction capabilities to increase the performance of the VANET environment.

## Figures and Tables

**Table 1:** Contributions and limitations of the existing approaches.

Approach	Contribution	Limitation
[24]	Utilizes blockchain to maintain privacy and security.	Requires computational and processing power to perform the operations effectively.
[25]	Utilizes trust, blockchain, and tendermint for security management.	Requires excessive energy.
[26]	Implements community architecture to manage trust among nodes.	Depends on fog nodes where communities may cause integrity challenges.
[27]	Uses Kalman filter technique to predict nodes’ behavior and prevent on-off attacks	Requires to be evaluated against other potential IoT attacks.
[28]	Utilizes dedicated networks for trust management for improving security.	Needs validation against existing potential attacks.

**Table 2:** Symbols and their description.

Symbols	Description	Symbols	Description
$\lim_{1.0}$	Limit to bound the absolute trust.	⊎	Binary operator used to perform addition.
∑	To formulate absolute trust value.	knw	Knowledge parameter of trust.
*p*	Trustor.	agt	Aggregation Process.
*r*	Trustee	pt	Past trust values.
*T*	Trust degree.	*n*	Number of observations available.
rep	Reputation component.	ab	Absolute trust.
ab	Ability parameter.	be	Benevolence parameter.

**Table 3:** Simulation Setup.

Parameters	Value
Area of network	200 m${}^{2}$, 300 m${}^{2}$, 400 m${}^{2}$
Number of nodes	50∼150 Nodes
Simulation time	50∼550 Min.
Transmission range	300∼350 m
Routing protocol	AODV, CBRD
MAC	IEEE 802.11
Mobility model	Random Direction Model
Transmission rate	6∼8 Mbps
Position of RSUs	x = 200, y = 200
Node Average Speed (Urban)	60∼90 km/h
Node Average Speed (Rural)	40∼60 km/h
